# A BCI System Based on Motor Imagery for Assisting People with Motor Deficiencies in the Limbs

**DOI:** 10.3390/brainsci10110864

**Published:** 2020-11-17

**Authors:** Omneya Attallah, Jaidaa Abougharbia, Mohamed Tamazin, Abdelmonem A. Nasser

**Affiliations:** Department of Electronics and Communications Engineering, College of Engineering and Technology, Arab Academy for Science, Technology and Maritime Transport, Alexandria P.O. Box 1029, Egypt; jaidaahany@hotmail.com (J.A.); tamazin@aast.edu (M.T.); menem_1954@yahoo.com (A.A.N.)

**Keywords:** brain–computer interface (BCI), motor Imagery (MI), electroencephalography (EEG), wavelet packet decomposition (WPD), feature selection (FS)

## Abstract

Motor deficiencies constitute a significant problem affecting millions of people worldwide. Such people suffer from a debility in daily functioning, which may lead to decreased and incoherence in daily routines and deteriorate their quality of life (QoL). Thus, there is an essential need for assistive systems to help those people achieve their daily actions and enhance their overall QoL. This study proposes a novel brain–computer interface (BCI) system for assisting people with limb motor disabilities in performing their daily life activities by using their brain signals to control assistive devices. The extraction of useful features is vital for an efficient BCI system. Therefore, the proposed system consists of a hybrid feature set that feeds into three machine-learning (ML) classifiers to classify motor Imagery (MI) tasks. This hybrid feature selection (FS) system is practical, real-time, and an efficient BCI with low computation cost. We investigate different combinations of channels to select the combination that has the highest impact on performance. The results indicate that the highest achieved accuracies using a support vector machine (SVM) classifier are 93.46% and 86.0% for the BCI competition III–IVa dataset and the autocalibration and recurrent adaptation dataset, respectively. These datasets are used to test the performance of the proposed BCI. Also, we verify the effectiveness of the proposed BCI by comparing its performance with recent studies. We show that the proposed system is accurate and efficient. Future work can apply the proposed system to individuals with limb motor disabilities to assist them and test their capability to improve their QoL. Moreover, the forthcoming work can examine the system’s performance in controlling assistive devices such as wheelchairs or artificial limbs.

## 1. Introduction

Motor deficits are a barrier for many paralyzed patients and people suffering from difficulty in moving their limbs. There are several causes of motor impairments such as spinal cord injury, a stroke of the motor cortex, neurodegeneration in the basal ganglia and cerebellum, or damage to the associational motor cortex. Spinal cord injury is mutilation to the spinal cord that leads to short-term or permanent alterations in its functions. Such alterations are developed deliberately in the sense of continuing neuroplasticity that reveals the damage of afferent response from the separated limbs [1]. Stroke is the foremost reason for long-term adult debility. It disrupts the brain’s blood supply, leading to an injury in brain tasks, specifically motor function. After a stroke, motor tasks’ impulsive response is related to activity feedback in the primary motor cortex [2].

On the other hand, Basal ganglia disease is a collection of mental complications that arise when the nuclei in the brain known as the basal ganglia defeat to restrain undesirable movements correctly or appropriately inform upper motor neurons circuits to recruit motor function [3]. Examples of Basal ganglia disease are Parkinson and Dystonia diseases. The cerebellar illness leads to incoordination, ineptness [4,5], and tremor as the cerebellum controls flattening and taming voluntary movements. It does not lead to the loss of any sole task but, conversely, produces an overall motor reaction [6]. Injury in various areas of the association cortex can generate higher-level syndromes of behavior. Apraxia is a condition of motor control that can happen after injury in the parietal association cortex, premotor cortex, or supplementary motor cortex. There is no disability of patient muscles or limbs in apraxia, and muscle power may not be reduced [7]. Motor deficiencies are affecting millions of people worldwide [8]. People with such disabilities experience an encumbrance in performing their daily functions. This dysfunction will decrease, and cease disable people’s daily routines and affect their quality of life (QoL) [9]. The QoL for those people can benefit from intelligent assistive technology that can enhance communication, house-environment arrangement, and motion, according to the individual’s enduring motor capabilities [10,11]. Thus, intelligent assistive systems such as brain–computer interface (BCI) are essential for helping those people achieve their daily actions and enhance their overall QoL [9]. Such a BCI system can use these people’s brain signals to control assistive devices to enable them to do their everyday tasks regardless of their motor disability.

BCI systems which are also known as the brain-machine interface (BMI) is a method used for controlling or communicating between a patient brain’s electrical activity and an external device such as a computer or robotic arm [11,12]. There are two ways to measure brain signals, namely invasive or non-invasive methods. Although the invasive method is very accurate, it requires a surgical operation to place the skull’s electrodes. On the other hand, the non-invasive approach is more comfortable in implementation and does not require any surgical intervention as only electrodes are attached to the scalp [13]. Among the non-invasive methods, electroencephalography (EEG) is a well-known technique that captures brain activity. Its popularity is due to the simplicity of use, mobility, lower cost, and high temporal resolution [14,15]. To produce an efficient and accurate BCI system capable of performing an intended task such as moving a hand, a foot, or a control wheelchair, significant features should be extracted from preprocessed EEG signals and then processed and analyzed. In BCI systems, extracted features are usually of high dimension. Therefore, the feature extraction process [16] is vital to pool out important information from EEG signals. Moreover, the features selection procedure is essential to reduce computational time and select a reduced number of features. Moreover, channel reduction is critical to lower the computational cost [17] further.

Motor imagery (MI) is when an individual internally imagine a part of his body is moving or performing an action. [18,19,20,21]. It can also be described as locomotion’s intellectual practice without any activation of the muscles [22]. This imagination of the motion produces a significant variation in the μ and β frequency bands’ amplitude in an individual’s EEG, recognized by the BCI system. This BCI system is beneficial for paralyzed people, as they are incapable of truly moving their limbs. However, they have kept the capacity to conceive the motion of paralyzed limbs. In this form, the BCI system can be used to control assistive devices such as prosthetic limbs [23]. Another sort of MI-based BCI system is spinal cord injury rehabilitation. Such a BCI system is connected to powered exoskeletons located on the paralyzed limbs. These exoskeletons use the EEG signal acquired during the MI task, which activates brain regions to enable individuals to move [23,24]. Several studies indicated the positive impact of BCI systems on the rehabilitation process [25,26]. EEG signals acquired during MI tasks are commonly used to constructed assistive BCI systems. A BCI system based on MI aims to get distinct EEG signals by imagining specific motor activities, which are then converted into actions [27,28]. Several BCI schemes based on MI have been proposed to help people with different motor disabilities attain a normal lifestyle [29,30]. Among these schemes, the studies [31,32] indicated that MI-based-EEG signals operated in BCI systems effectively treat patients with spinal cord injury. Also, the articles [22,33,34] verified that MI has a constructive influence on motor rehabilitation after stroke. Since the execution of MI is interior to the patient, and therefore not directly noticeable, BCI can ease the MI-based stroke rehabilitation by providing a straight and instant response on the MI performance [22]. Moreover, the authors in [35] proved that MI could activate neuroplasticity in ipsilesional motor cortical areas despite acute paralysis after stroke.

The authors in [28] introduced a system that allowed paralyzed patients to control wheelchairs by imagining the right and left hands and feet’ movement. The authors first used surface Laplacian derivation (SLD) for spatial filtering. Then they used the Mahalanobis linear distance (MLD) classifier for classification. The authors in [36] proposed a BCI system to help the paralyzed patients control artificial limbs. In [37], the authors designed a BCI system to control a real-time wheelchair. The authors in [38] built a BCI system that successfully integrates a wheelchair with artificial limbs. They used the common spatial patterns (CSP) method to extract features and an SVM classifier. Although the system proposed in [38] was built to perform an everyday task, no feature selection was used to reduce the execution time. Edla et al. [39] proposed a system to control wheelchairs based on wavelet packet decomposition (WPD). Another approach was suggested by Roy et al. [40] to control artificial limbs. The authors in [41] proposed a system depending on spatial-frequency temporal domains to extract new informative features. The classification process was done using a sparse representation classifier (SRC). The BCI competition III–IVa dataset [42] was used later by Singh et al. [43] who proposed a BCI system that is not patient dependent. The authors of this study applied a spatial filter to reduce the data dimension.

Then using known channels, they extracted information features using the symmetric positive definite (SPD) method. Selim et al. [44] extracted features using CSP. Moreover, the authors proposed a novel feature selection model that reduces the number of extracted features. Also, they applied segmentation to remove epochs during resting time. In [45], Yuan et al. used frequency domain features by applying the Fourier transform (FT) for MI-based BCI. The authors in [46] extracted features using a time domain, including the root mean square (RMS) method. The authors compared the performance of the proposed system with different channel sets. A summary of recent related BCI systems is shown in Table 1.

This study proposes a novel efficient BCI system based on MI to differentiate between different limb motor tasks. Another goal is the make the system easier to set up. To achieve these goals, we proposed a novel system to investigate a different combination of channel sets previously used in the literature instead of using the whole number of channels available in the dataset. These channels sets were used separately in different research articles and were verified to produce efficient results. However, we examined which set has the highest performance using the hybrid feature set proposed in the paper. Second, to further reduce the number of channels, we proposed a hybrid feature selection approach that selects the most significant features that impact the BCI performance. These reduced selected features will consequently reduce the number of channels. The performance of the proposed BCI to classify MI tasks with high accuracy is tested. The possibility of reducing the number of features and channels while improving classification accuracy is also examined. The system may be considered an initial step for achieving a complete real-life--based framework to assist people with motor disability. The proposed BCI uses such people’s EEG signals to control assistive devices such as wheelchairs or wheelchairs regardless of their motor deficiency. To overcome the challenges from previous studies that used either time, frequency, or time-frequency feature extraction methods, the proposed system introduces a hybrid feature extraction used to classify MI tasks. Also, to reduce the computational cost of the BCI system, which was overlooked in previous studies, we present a feature selection procedure to determine the most significant features that are capable of differentiating between right hand and foot motor imagery tasks based on the μ frequency range (8–13 Hz) and β frequency range (13–30 Hz) of EEG signals [47]. Also, different combinations of channels are investigated to select the combination, which has the highest impact on the proposed BCI system’s performance. Finally, three machine-learning classifiers are used to translate human brain EEG signals to commands or actions. To test the proposed BCI system’s efficacy, two public datasets are used, specifically, the BCI competition III–IVa dataset and the autocalibration and recurrent adaptation dataset.

**Table 1:** Literature review of current BCI systems.

Article	Application	Feature Extraction	Classifier	Disadvantages	Accuracy (%)
[27]	Limb Motor Task	Spatial-frequency-temporal patterns	SRC	High computational cost	75.46
[46]	Limb Motor Task	RMS	LDA	Time-domain features not suitable for analysis EEG datasets	78.77
[38]	Integrate a wheelchair and an artificial limb	CSP	SVM	CSP suffers from degradation in performance in case of non-Gaussian distributions	80
[44]	Limb Motor Task	CSP	SVM	Required high input channels	85.01
[29]	Limb Motor Task	CSP	MDRM	Required high input channels	86.13
[40]	Control an artificial limb	DWT ^1^	LDA QDA KNN	DWT gives lower frequency resolution than WPD	86.9
[36]	Control artificial limb by paralyzed patients	HOS ^2^ + DWT	LDA	DWT gives lower frequency resolution than WPD	89.5
[39]	control wheelchairs	WPD	SVM	High computational cost	90.698
[45]	Limb Motor Task	FT	MNFD	FT not suitable for analysis of EEG data	90.89
[28]	Limb Motor Task	CSP/CSP + AR ^3^	LDA	Patient dependent and cannot be generalized on all patients in any dataset	
[23]	Real-time wheelchair control	FFT ^4^	FNN	FFT not suitable for analysis EEG dataset	92.0
[48]	Limb Motor Task	DWT + WPD	KNN	KNN suffers from the curse of dimensionality	92.8
[28]	2-D virtual wheelchair control	SLD	MLD	Spatial filters such as SLD may destroy features‘ information	98.4

^1^ DWT is the discrete wavelet transform, ^2^ HOS is higher-order statistics, ^3^ AR is autoregression, ^4^ FFT is the fast Fourier transform.

This paper’s remainder is organized as follows: In Section 2, we describe the two datasets used and provide details for the proposed BCI model. In Section 3, we present the performance evaluation metrics, and we demonstrate the results in Section 4. In Section 5, we discuss the significance of the proposed FS on the proposed BCI’s computational cost and compare the proposed BCI system’s performance with previous studies. Finally, we provide some conclusive remarks for this study in Section 6.

## 2. Materials and Methods

### 2.1. Datasets

We used two publicly available datasets to test the effectiveness of the proposed BCI system. These datasets contain EEG signals collected during MI tasks. The two datasets are the BCI competition III–IVa dataset [42] and the autocalibration and recurrent adaptation dataset [49]. Details for these datasets are included in Appendix A and Appendix B, respectively.

### 2.2. The Proposed BCI System

**Figure 1:**
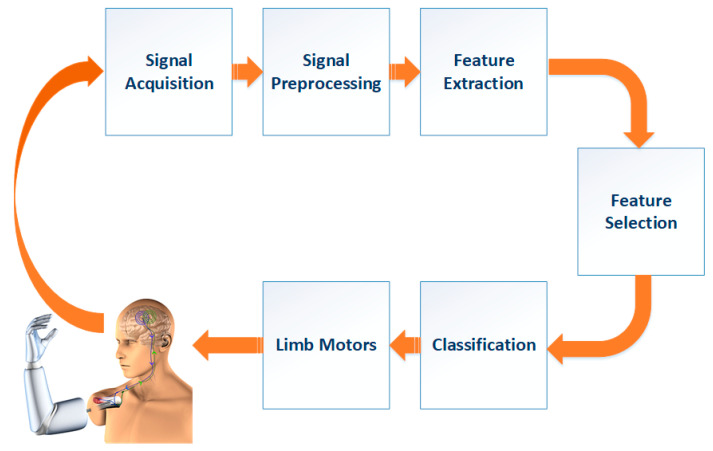
The proposed BCI system.

This study proposes a novel BCI system based on MI to classify different limb motor tasks. The proposed system’s key objective is to construct a portable and easy to set up EEG-BCI based on motor-imagery training neuro-feedback system to classify motor-imagery tasks in real time with high accuracy. The proposed system can be supposed as a primary stage for attaining an entire real-life-based framework to assist individuals with motor debility. The proposed BCI system consists of the following steps: signal preprocessing, feature extraction, feature selection, and finally, classifying features. These steps are presented in Figure 1.

#### 2.2.1. Preprocessing

Noise removal is necessary for EEG. Preprocessing is used to enhance noisy signals and remove artifacts. Sources of noise and artifacts may include power-line interference, electrocardiography (ECG), electromyography (EMG), electrooculogram (EOG), and individuals’ movements [14,50]. In this study, the preprocessing phase is divided into four steps. In the first step, a bandpass filter is used. The two EEG frequency sub-bands (μ and β rhythms) are extracted from the EEG signals by cutting the frequency from 10 Hz to 30 Hz and removing all the remaining range. The bandpass filter is implemented using a 5th order Butterworth finite impulse response (FIR) filter. Afterwards, the input signal is normalized to remove the absolute amplitude and keep the relative amplitudes. Normalization significantly reduces the channel dispersion in comparison with non-normalized recordings. Both datasets are normalized using Equation (1).

(1) $$z_{i}=\frac{w_{i}-\bar{w}}{\delta }$$

where $z_{i}$ is the normalized EEG signal, $w_{i}$ is the original EEG signal, $\bar{w}$ and δ are the mean and standard deviation, respectively.

In the third step, a notch filter is applied to eject the power-line interference at 60 Hz from the BCI competition III–IVa dataset’s EEG signals. Please note that a notch filter was already applied to the autocalibration and recurrent adaptation dataset according to that dataset’s owners. Lastly, in the fourth step, EEG signals are segmented to extract the epochs with windows from [0.5 to 3.5] s duration for the BCI competition III–IVa dataset and from [3 to 8] s duration for the autocalibration and recurrent adaptation dataset.

#### 2.2.2. Feature Extraction

One of the challenges in any BCI system is to extract the most representative features capable of distinguishing between different MI tasks from the processed EEG signals. Motor activities are then translated into real actions. Useful features lead to an accurate BCI system; therefore, we present two feature sets in this study. The former is extracted in the time domain, whereas. The second is extracted in the time-frequency domain. Afterwards, the two feature sets are combined to form a hybrid feature set to test feature fusion’s influence on the proposed BCI [51]. The details of feature extraction methods are presented in this section.

(1) Feature extraction in the time domain (Feature Set 1)

Time-domain features are directly extracted from the processed signal. In this study, six features are extracted in the time domain, including RMS, Renyi entropy, and Hjorth parameters, including the following parameters: (activity, mobility, complexity), wavelength, and mean absolute value.

i. Root Mean Square

RMS is the square root of the average of the signal’s squared value in the time domain and can be represented with the following equation [46].

(2) $$\mathrm{RMS}=\sqrt{\frac{1}{M}\overset{M}{\underset{j=1}{\sum }}y_{j}{}^{2}}$$

ii. Renyi Entropy

Renyi uses brain activity to identify the complexity of time series. Then, the disorder in the BCI system, as illustrated in the following equation [46]:

(3) $$\mathrm{Renyi}=\log\frac{\sum_{j=1}^{M}y_{j}{}^{q}}{1-q}$$

iii. The Hjorth parameter [52]

The Hjorth parameters include the complexity and mobility parameters.

(4) $$\mathrm{mobility}=\sqrt{\frac{var\left(y_{j}^{\prime }\right)}{var\left(y_{j}\right)}}$$

(5) $$\mathrm{Complexity}=\frac{\mathrm{mobility}\left(y_{j}^{\prime }\right)}{\mathrm{mobility}\left(y_{j}\right)}$$

iv. The waveform length [53]

Waveform length (WL) measures the signal complexity

(6) $$\mathrm{WL}=\overset{M}{\underset{j}{\sum }}\left|y_{j}-y_{j-1}\right|$$

v. The mean absolute value [48]

(7) $$\mu =\frac{1}{M}\overset{M}{\underset{j=1}{\sum }}\left|y_{j}\right|$$

where M is the number of samples in each sub-band, $y_{j}${*y_j_*_= 1_, *y_j_*_= 2_, …, *y_j_*_= *M*_} are the samples in the time domain, and the ‘q’ parameter controls the shape of the probability distribution, $var\left(y_{j}^{\prime }\right)$ is the variance of the first derivative of the signal sample *y_j_*, $var\left(y_{j}\right)$ is the variance of the signal sample *y_j_*, $y_{j}\;\mathrm{and}\;y_{j-1}$ are the current and previous samples in the time domain,$\mathrm{mobility}\left(y_{j}^{\prime }\right)$ is the mobility of the first derivative of the signal sample *y_j_*, $\mathrm{mobility}\left(y_{j}\right)$ is the mobility the signal sample *y_j_*, $\left|y_{j}\right|$ is the absolute value of the sample *y_j_*.

(2) Feature extraction in the time-frequency domain (Feature Set 2)

FT and linear models have been commonly used to analyze the EEG signals; however, the analysis using time-frequency models achieved remarkable success over FT [54,55,56]. Among the various wavelet transform (WT) methods, the discrete wavelet transform technique (DWT) decomposes the signal in the time-frequency domain to approximation coefficients, representing the low pass filter and the detail coefficients, which represent the high pass filter. For multi-stage decomposition, only the approximate components are further decomposed, followed by down-sampling by 2. On the other hand, the WPD is an improvement or extension to the DWT. For multi-level decomposition, WPD decomposes both the approximation (lowpass) and detail (highpass) [57]. WPD produces $2^{j}$ sets of wavelet coefficients and gives a better time-frequency resolution for the decomposed signal more than the DWT [58].

In this study, four levels of WPD are used to decompose signals and then four different features are extracted from the decomposed signals. In addition to two HOS features [48].

i. The absolute mean of coefficients in each sub-band (*μ*)

(8) $$\mu =\frac{1}{N}\overset{N}{\underset{i=1}{\sum }}\left|x_{i}\right|$$

ii. The average power of coefficients in each sub-band (*P_av_*)

(9) $$P_{av}=\sqrt{\frac{1}{N}\overset{N}{\underset{i=1}{\sum }}x_{i}{}^{2}}$$

iii. The standard deviation of the coefficients in each sub-band (σ)

(10) $$\sigma =\frac{1}{N}\overset{N}{\underset{i=1}{\sum }}{\left(x_{i}-\mu \right)}^{2}$$

iv. The ratio of the absolute mean values of coefficients of adjacent sub-bands (γ)

(11) $$\gamma =\frac{\frac{1}{N}\sum_{i=1}^{N}\left|x_{i}\right|}{\frac{1}{N}\sum_{i=1}^{N}\left|z_{i}\right|}$$

v. The Skewness of the coefficients (signal) in each sub-band (*S*)

(12) $$S=\frac{1}{N}\overset{N}{\underset{i=1}{\sum }}\frac{{\left(x_{i}-\mu \right)}^{3}}{\sigma^{3}}$$

vi. The Kurtosis of the coefficients (signal) in each sub-band (*K*)

(13) $$K=\frac{1}{N}\overset{N}{\underset{i=1}{\sum }}\frac{{\left(x_{i}-\mu \right)}^{4}}{\sigma^{4}}$$

where N is the cue’s length in each sub-band, and *X*{*x*_1_, *x*_2_, … *x_N_*} and *Z*{*z*_1_, *z*_2_, …, *z_N_*} are two adjacent sub-bands after WPD.

Finally, the two feature sets are combined and used to test the impact of the feature fusion process on the proposed BCI system’s performance.

#### 2.2.3. Feature Selection

The hybrid feature set extracted in the feature extraction stage is of large dimension. Such a large dimension increases the BCI system’s classification step’s complexity and usually reduces its performance. Therefore, feature selection (FS) is essential. FS is commonly used in medical systems to decrease the feature set’s size and omit excessive and irrelevant features [59]. Feature selection is divided into three main categories which are: filter, wrapper, embedded, and hybrid. The filter FS method is the easiest and quickest method. Filter FS employs a metric for selecting features. The main drawback of such a technique is the independence of the classification process.

On the other hand, the wrapper FS is dependent on the classification procedure, but it is more complicated and slower than the previous filter method. In the embedded method, the FS process is inserted within the classifier structure. The embedded FS technique comprises the interface within the classification process. It requires less execution time than the wrapper FS method. Finally, the hybrid method fuses two or more of the previous FS methods, which usually enhances the BCI system’s performance.

For this reason, we use a hybrid feature selection method in this study, where filters and wrappers methods are combined. Most significant features are selected at the beginning of the available feature set via the computationally efficient Correlation-based Feature Selection (CFS) filter method [60]. For more refinement, the classifier subset evaluator wrapper method [51], which includes SVM, LDA, and KNN classifiers, is used to select significant features. We applied a method where the filters and wrappers are merged to select the most notable features among the ranked features.

**Correlation-based Feature Selection (CFS**) is a well-known filter FS method which measures the similarity between two features. If two features are correlated, the correlations coefficients value will be between (−1 to 1), and if the two features are not correlated, they will have a correlation coefficient near 0. **Classifier Subset Evaluation (CSE)** is one of the most commonly known wrapper feature selection methods. It uses a classifier to estimate the ‘merit’ of a set of attributes. This feature subset is used to train a classifier, and the accuracy of that classifier determines its effectiveness. The classifier subset evaluator avoids overfitting by using cross-validation measures of predictive accuracy. In this study, a forward stepwise searching strategy method is used where the model starts with no features, then iteratively adds features that improve the model performance. Once additional features no longer improve the classification accuracy, the CSE method will not add any more features to the subset [61].

#### 2.2.4. Classification

All feature combinations on the classification accuracy are tested on three different classifiers, specifically, SVM, LDA, and KNN. Although every subject in the dataset contains separate train and test sets, they are combined into one dataset due to the low number of trials. A 10-fold cross-validation (CV) approach is employed to validate the results where the dataset is randomly split into ten different sets with equal sizes. The ten groups consist of nine training sets and one testing set. The model is trained using the nine different training sets and tested using the testing set every round. The classification accuracy (CA) of the testing set is then calculated. This process is repeated ten times, and the average accuracy of all rounds are calculated. In this study, a linear kernel was used for the SVM classifier. For the k-NN, the Euclidean distance was used. The three classifiers used in this study were implemented in MATLAB using the X, Y, Z libraries/packages versions X, Y, Z [50,62,63].

## 3. Performance Evaluation

Numerous metrics are used to assess the performance of the proposed BCI system. These metrics are the CA, sensitivity, specificity, F1-score, precision, and receiver operating characteristic curve (ROC) [14]. Equations (14)–(18) are used to calculate these metrics.

Accuracy is an evaluation metric used to determine how the entire data classifier has correctly classified many motor tasks. Therefore, it specifies the capability of the classifier to execute well.

(14) $$\mathrm{Accuracy}=\;\frac{\mathrm{TP}+\mathrm{TN}}{\mathrm{TP}+\mathrm{TN}+\mathrm{FP}+\mathrm{FN}}$$

(15) $$\mathrm{Sensitivity}=\frac{\mathrm{TP}}{\mathrm{TP}+\mathrm{FN}}$$

(16) $$\mathrm{Specificity}=\frac{\mathrm{TN}}{\mathrm{TN}+\mathrm{FP}}$$

where TP, FN, TN, and FP represent the true positive, false negative, true negative, and false-positive rates, respectively.

Precision is calculated as the ratio of correctly predicted positive examples divided by the summation of true positive and false-positive predictions.

(17) $$\mathrm{Precision}=\frac{\mathrm{TP}}{\mathrm{TP}+\mathrm{FP}}$$

The F1-score is calculated to evaluate the system performance. Classification accuracy is commonly used as it is a single measure used to summarize model accomplishment. F-Measure provides a way to combine both precisions and recall into a single measure and captures both properties.

(18) $$F1-\mathrm{Score}=\frac{\left(2\times \mathrm{Precision}\times \mathrm{Recall}\right)}{\mathrm{Precision}+\mathrm{Recall}}$$

The area under the receiver operating characteristic curve (AUC) is a method for evaluating models based on each point’s average on the ROC curve. The ROC curve is a plot of the *true positive rate* against the *false-positive rate*. The AUC is the area under this curve, and its value is always between 0 and 1. For a given classifier, a higher AUC value indicates a better classifier performance.

## 4. Experimental Results

This study aims to construct an effective BCI system based on MI to distinguish amid several limb motor tasks. Moreover, develop a portable and affordable EEG-BCI based on MI training neuro-feedback system to classify motor-imagery tasks in real time with high accuracy. The presented BCI system could be deemed a former phase for accomplishing a comprehensive real-life--based framework to help people with motor deficiencies. To achieve this goal, we present a new BCI system that consists of four experiments. Reducing the number of EEG channels used in MI tasks classification would make the BCI system more mobile and easier to set up, and maintain a real-time EEG-based BCI system. Therefore, the four experiments for the proposed BCI system are carried out on different combinations of channel sets to determine the significance of channel reduction and select the channel set, which has a higher impact on the BCI system’s performance. For the BCI competition III–IVa dataset, the full set of channels is 118 channels. Three combinations of channel sets are used. The first channel set includes the C3, Cz, and C4 channels suggested by S. Selim et al. in [35] and is known as “Channel set 1 BCI III”. The second channel set contains 18 electrodes around the sensorimotor cortex in the β frequency range, including the channels “C5, C3, C1, C2, C4, C6, CP5, CP3, CP1, CP2, CP4, CP6, P5, P3, P1, P2, P4, and P6” and it was suggested by Wang et al. [64] and known as “Channel set 2 BCI III”. In addition, finally, the third channel set further, which includes the 25 channels on the parietal lobe that was suggested in [35] and named “Channel set 3 BCI III”. On the other hand, for the autocalibration and recurrent adaptation dataset, two-channel sets were suggested. The first channel set consists of 13 channels, including C3 (FC3, C5, CP3, and C1), Cz (FCz, C1, CPz, and C2) and C4 (FC4, C2, CP4, and C6) and is named “Channel set 1 Auto”. The second channel set consists of 3 channels, C3, Cz and C4 and are called “Channel set 2 Auto”.

**Figure 2:**
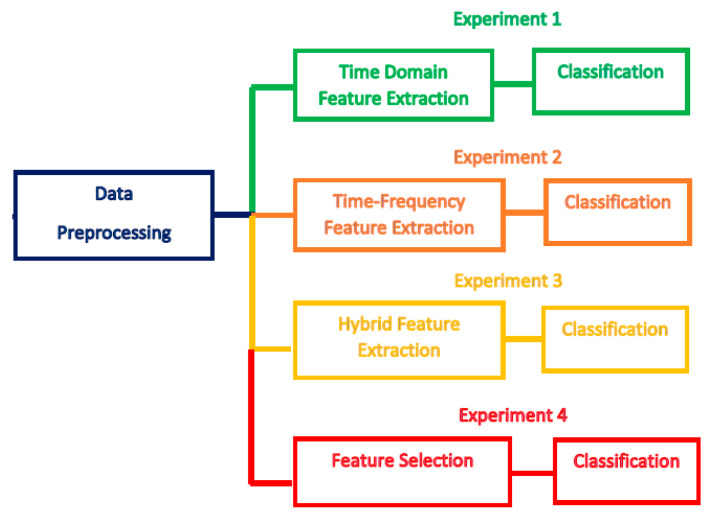
The four experiments of the proposed BCI system.

Figure 2 illustrates the four experiments conducted in this study that are constructed with different channel sets. The four experiments are summarized as follows:

Experiment 1—six time-domain features are extracted and used for classifying MI tasks.
Experiment 2—six time-frequency features are extracted and used for classifying MI tasks.
Experiment 3— a hybrid feature set is used for classifying MI tasks.
Experiment 4—a hybrid FS is employed to select the most relevant features and classify MI tasks.

### 4.1. Experiment 1—Time-Domain Features (Feature Set 1)

The results of Experiment 1 are discussed in this section for BCI competition III–IVa and autocalibration and recurrent adaptation datasets.

#### 4.1.1. BCI Competition III–IVa Dataset Results

**Figure 3:**
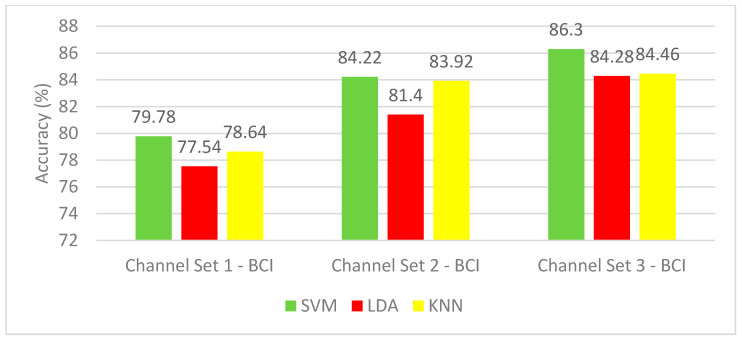
Experiment 1—Time-domain features—Classification Accuracies of BCI competition III–IVa dataset.

This section covers the effect of using only time-domain features on the classification of mental motor-imagery tasks using the BCI competition III–IVa dataset. Figure 3 shows the mean classification accuracies for the five subjects of the dataset using SVM, LDA, and KNN classifiers constructed with the three-channel sets. Figure 3 can clearly show the superiority of the channel set 3 BCI (25 channels) set over the two other electrodes sets. This is because the SVM classifiers achieved mean accuracies of 79.78% and 84.22% using the channel set 1 BCI (3 channels) and channel set 2 BCI (18 channels), respectively, which are lower than the mean CA of 86.3% achieved by the SVM classifier constructed using the channel set 3 BCI. The LDA and KNN classifiers constructed with channel set 1 BCI attained 77.54% and 78.64%, respectively. However, when using the SVM classifier, a mean accuracy of 79.78% is reached. Mean accuracy of 81.4% and 83.92% is obtained using the LDA and KNN classifiers constructed with channel set 2 BCI, whereas using the SVM classifier, an accuracy of 86.3% is reached. On the other hand, the KNN classifier constructed with channel set 3 BCI (25 channels) reached a mean CA of 84.46%, which is lower than the SVM (86.3%) constructed with the same channel set. The results of Experiment 1 show that the highest mean accuracy is achieved with the SVM classifier (86.3%) built with channel set 3 BCI (25 channels).

#### 4.1.2. Autocalibration and Recurrent Adaptation Dataset Results

**Figure 4:**
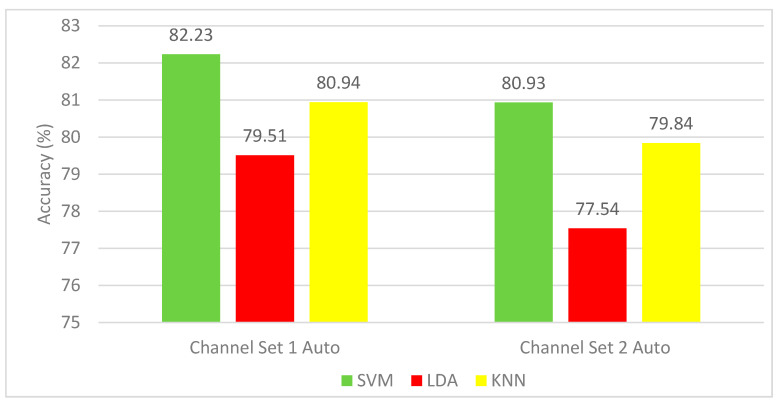
Experiment 1—Time-domain features—Classification Accuracies (%) autocalibration and recurrent adaptation dataset.

The results of Experiment 1 for the autocalibration and recurrent adaptation dataset are shown in Figure 4. This figure shows a comparison between SVM, LDA, and KNN classifiers using the channel set 2 Auto (3 channels) and channel set 1 Auto (13 channels). It is evident from Figure 4 that the SVM classifier shows a remarkable success over the other two classifiers. It is clear that the mean CA (82.23%) for all subjects for SVM constructed using the channel set 1 Auto (13 channels) is higher than that of the SVM (80.93%) constructed using the channel set 2 Auto (13 channels).

### 4.2. Experiment 2—Time-Frequency Domain Features (Feature Set 2)

#### 4.2.1. BCI Competition III–IVa Dataset Results

**Figure 5:**
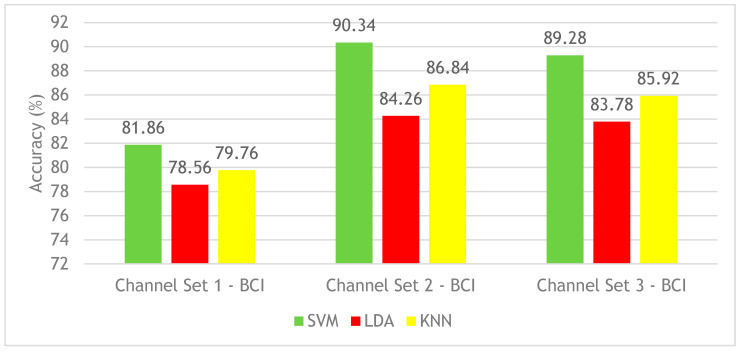
Experiment 2—Time-Frequency domain—Classification Accuracies—BCI competition III–IVa dataset.

This section discusses the results of Experiment 2 on the BCI competition III–IVa dataset. Figure 5 shows the CA results for SVM, LDA, and KNN classifiers constructed using the three different channel sets’ time-frequency features. Figure 5 shows that the SVM classifier achieved the highest mean CA among classifiers. The mean CA using SVM constructed with channel set 1 BCI (3 channels) is 81.86%. However, channel set 2 (18 channels) achieved a mean CA of 90.34% 12, which is higher than the mean CA (89.28%) performed using the channel set 3 BCI (25 channels) and with 13 channel set 1 BCI (3 channels). It is clear from Figure 5 that the time-frequency features have increased the CA of the three classifiers for the three combinations of channel sets compared to Experiment 1 results (see Figure 3). Although the performance of the proposed BCI system constructed with time features has shown the highest CA of 86.3% (Experiment 1) when applying the channel set 3 BCI (25 channels set), the time-frequency features (Experiment 2) enhanced the CA. The highest CA of 90.4% is achieved with channel set 2 BCI (18 channels), both using an SVM classifier.

#### 4.2.2. Autocalibration and Recurrent Adaptation Dataset Results

**Figure 6:**
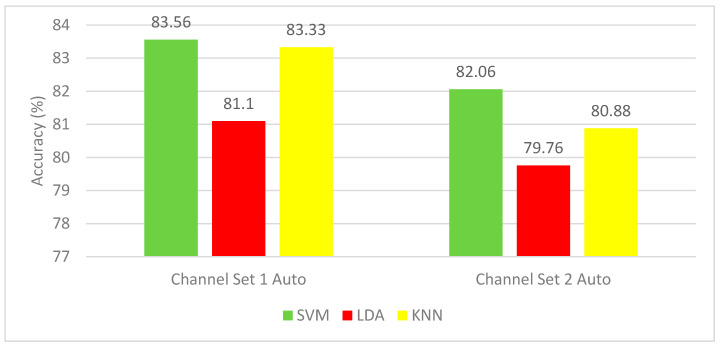
Experiment 2—The Time-Frequency domain features’ classification accuracies for the autocalibration and recurrent adaptation dataset.

The results of Experiment 2 for the autocalibration and recurrent adaptation dataset are shown in Figure 6. This figure shows a comparison between SVM, LDA, and KNN classifiers using the channel set 2 Auto (3 channels) and channel set 1 Auto (13 channels). SVM classifier shows the highest mean CA for all subjects. SVM classifier achieved a mean CA of (83.56%) for all subjects using the channel set 1 Auto (13 channels), which is higher than that of the SVM (82.06%) constructed using the channel set 2 Auto (3 channels).

### 4.3. Experiment 3—Hybrid Features

#### 4.3.1. BCI Competition III–IVa Dataset Results

**Figure 7:**
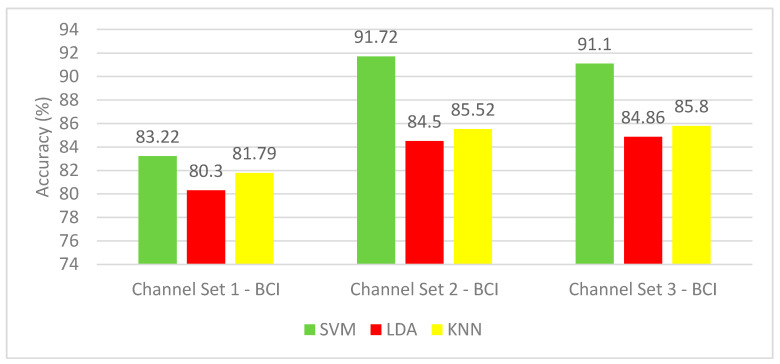
Experiment 3—The Hybrid features’ classification accuracies for BCI competition III–IVa dataset.

The results of the hybrid feature set for the BCI competition III–IVa dataset are shown in Figure 7. The mean CA for channel set 2 BCI (18 channels) outperforms the other channels’ accuracy for the three classifiers. Channel set 1 BCI (3 channels) showed the lowest performance with mean CA range (80.3–83.22%), whereas channel set 2 BCI (18 channels) showed the highest mean CA range (84.5–91.72%). The SVM classifier achieved the most elevated mean CA for the three-channel sets configurations. In contrast, the LDA classifier yielded the worst classification accuracy results among the three classifiers. The highest mean CA of 91.72% is achieved using an SVM classifier constructed with channel 2 BCI (18 channels).

Once a model is constructed, it is crucial to decide whether it is adequate to make robust predictions. CA alone is typically not enough metric to make this decision. Therefore, sensitivity, specificity, precision, F 1-score, and area under ROC curve (AUC) have been calculated for all subjects to give a clear and accurate evaluation of the proposed system and are shown in Table 2. All the mentioned parameters have been measured for SVM classifiers constructed with hybrid features of channel set 2 BCI (18 channels) as it showed the highest performance in Table 2. Subject “al” achieved the most heightened sensitivity, specificity, and precision. The highest F1-score and AUC of 0.989 and 1 were performed by subject “al,” as well.

#### 4.3.2. Autocalibration and Recurrent Adaptation Dataset Results

**Figure 8:**
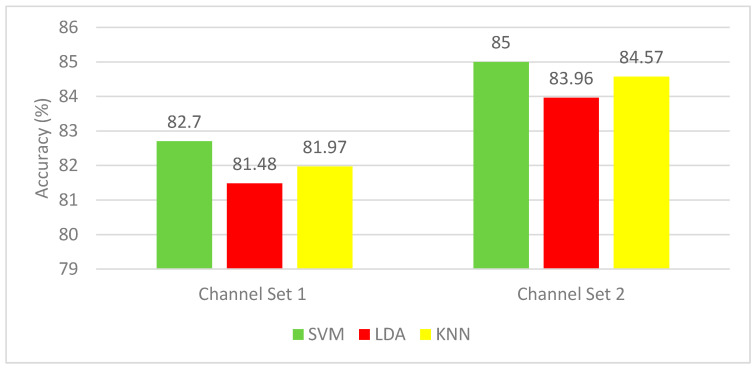
Experiment 3—Hybrid features—Classification Accuracies for the autocalibration and recurrent adaptation dataset.

In this section, the results are presented and discussed for the autocalibration and recurrent adaptation dataset. Figure 8 shows a comparison between CAs of SVM, LDA, and KNN for the two-channel sets. Channel set 1 Auto includes 13 channels, whereas channel set 2 consists of 3 channels. Each subject performed two or three runs. Figure 8 indicates that the SVM classifier has superior performance with CA ranging from 82.76%–85%, which is higher than that of the K-NN (81.79%–84.57%), as well as the LDA (81.48%–83.96%).

The sensitivity, specificity, precision, F1-score, and area under the ROC curve (AUC) metrics are calculated for all subjects to give a clear and accurate evaluation of the proposed system (Table 3). These metrics have been measured for SVM classifiers constructed using hybrid features of channel set 1 Auto (13 channels) since it showed the highest performance (Figure 8). It is evident that the first subject’s first-run “S01A” yielded the most heightened sensitivity, specificity, precision, and F1-score. The AUC for all subjects’ runs was equal.

### 4.4. Experiment 4—Feature Selection for Hybrid Features

#### 4.4.1. BCI Competition III–IVa Dataset Results

This section covers using the introduced FS on the proposed BCI system’s performance using the BCI competition III–IVa dataset. Table 4 shows the CA and the mean CA for the five subjects of the dataset using SVM, LDA, and KNN classifiers constructed using the channel set 2 BCI (18 channels) since this channel set achieved the highest performance in Experiment 3.

In Table 4, we compare the performances of the three classifiers after applying the presented FS technique. We show that FS improves the CA from 91.72% (the highest CA achieved in Experiment 3) to 93.46% using Linear-SVM, as shown in Table 4 (an improvement of 1.74%). Also, for LDA and K-NN classifiers, the CAs have improved by 2.23% and 3.12%, respectively.

**Figure 9:**
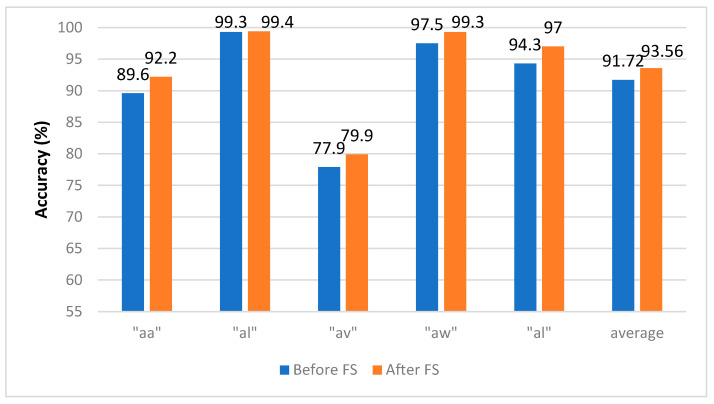
Experiment 4—The Hybrid Feature Selection using the SVM classifier constructed using the channel set 2 BCI. for the BCI competition III–IVa dataset.

Figure 9 shows the CA for Experiment 4 using the SVM classifier constructed with hybrid features using the channel set 2 BCI (18 channels), which showed its superiority in Experiment 3. In this figure, we compare the subject CA before and after applying FS. The average CA increased from 91.72% to 93.56% using the channel set 2 BCI (18 channels).

In Table 5, we show the performance metrics for the SVM classifier constructed with the selected features of the hybrid feature set using the channel set 2 BCI (18 channels) for the BCI competition III–IVa dataset. The proposed FS has enhanced sensitivity, specificity, precision, F1-score, and AUC. For example, sensitivity for subject “aa” has increased from 0.907 to 0.914, and the specificity has increased from 0.893 to 0.907 after feature selection. On the other hand, the precision and F1-score for subject “av” have risen from 0.705 and 0.720 to 0.780 and 0.783, respectively.

#### 4.4.2. Autocalibration and Recurrent Adaptation Dataset Results

This section describes the effect of using the introduced FS on the proposed BCI system’s performance using the autocalibration and recurrent adaptation dataset. Table 6 shows the CA and the mean CA for the 12 subjects of the dataset using SVM, LDA, and KNN classifiers constructed with the channel set 1 Auto (13 channels). This channel set achieved the highest performance in Experiment 3. Table 6 shows that the mean CA for all subject using SVM classifier reached 86.41% instead of 85.0% without FS (Experiment 3). The bold values in Table 6 represent the highest CA achieved using the SVM classifier for each subject individually and all subjects. The SVM classifier showed better performance for all subjects except for subject S01. S01 had an average CA of 99.5% when using K-NN and 99.25% for the Linear-SVM.

**Figure 10:**
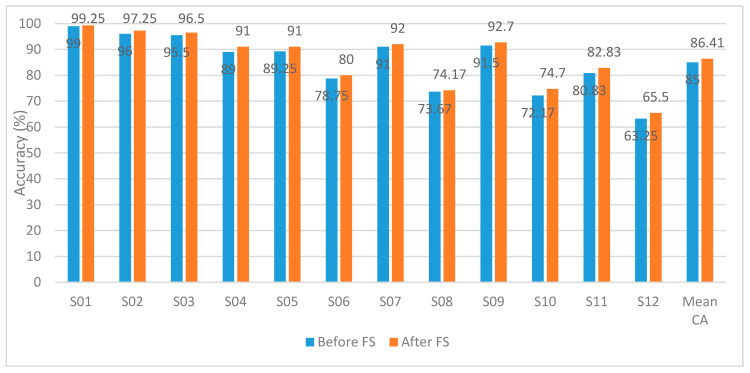
Experiment 4—Hybrid Feature Selection using SVM for the autocalibration and recurrent adaptation dataset–Channel Set 1 Auto.

In Figure 10, we compare using the hybrid features extracted from the channel set 1 Auto before and after applying feature selection techniques using the SVM classifier. The results emphasize the importance of applying feature selection on each subject. After applying feature selection, CA has increased for all subjects. The mean CA has increased from 85 to 86.41% using the channel set 1 Auto.

Table 7 shows the SVM classifier’s performance metrics constructed with selected features of hybrid feature set using the channel set 1 Auto (13 channels) for autocalibration and recurrent adaptation dataset. Applying the proposed FS technique has enhanced sensitivity, specificity, precision, F1-score, and AUC. For example, sensitivity for subject “S01A” has increased from 0.936 to 0.957, and the specificity has increased from 0.929 to 0.950 as well, after feature selection. Also, the precision and F-Measure for subject “S02A” have increased from (0.705 and 0.720) to (0.780 and 0.783), respectively.

## 5. Discussion

This study proposes a novel efficient BCI system based on EEG data collected while performing MI tasks. The proposed system’s primary goal is to construct a portable and low-cost BCI based on MI training neuro-feedback system to classify limb MI tasks in real time with high accuracy. The proposed system is an initial step for developing a comprehensive real-life--based framework to help people with motor deficiencies to perform their daily activities. The evaluation of the proposed BCI system consists of three components. Lowering the number of EEG channels employed in the BCI system construction can lead to a more portable and easier system to set up. It also maintains a real-time EEG-based BCI system. Thus, the system’s three evaluation metrics are based on different combinations of channels set to determine the significance of channel reduction and then select the channel set with the strongest influence on the BCI system’s performance. We first compare two feature sets. The first feature set consists of statistical time features, whereas the second one consists of time-frequency features. Second, we examine the influence of fusing time and time-frequency features (hybrid feature set). The results suggest that the combination of time and frequency features increases the proposed BCI system’s performance. To create an efficient BCI system, the computational system cost should be lowered, and this could be done by selecting a reduced number of significant features. Lastly, we used an FS approach to reduce the feature set, which impacts the proposed BCI system’s accuracy.

To further evaluate the performance of the proposed FS (Experiment 4), two more parameters were considered: the number of features selected and the execution time before and after FS, as shown in Table 8 for BCI competition III–IVa and autocalibration and recurrent adaptation datasets. The results in Table 8 indicate that FS significantly decreases the BCI competition III–IVa dataset’s execution time. Moreover, the number of features was reduced. For channel set 2 (the 18 channels configuration), the average number of features was reduced from 1836 features per run to 6 features. Moreover, the average execution time decreased from 13.1964 s to 1.2694 s. FS has significantly reduced the execution time for the autocalibration and recurrent adaptation dataset as well. Also, the number of features was reduced, and the average number of features decreased from 1326 features per run to 9.679 features per run. The average execution time was also reduced from 12.6015 s to 1.8571 s.

To verify our proposed system’s effectiveness, the results were compared with the classification accuracy of recent related studies (Tables S7 and S8). In Table S7, we compare the CA of the proposed BCI system and recent studies based on the BCI competition III–IVa dataset. Although Wang et al. method [64] obtained an accuracy of 94.2%. This CA is patient dependent and is not generalizable to all patients. This is because the authors used three different feature extraction methods that are dependent on the patient. For ‘al,’ ‘aw,’ and ‘ay,’ they used the CSP algorithm on Event-Related Desynchronization (ERD). The remaining subjects used the hybrid feature set (CSP and Autoregressive (AR)) extracted from 18 channels. Singh et al. [43] designed a spatial filter which reduces the dimension of Sample Covariance Matrices. The authors achieved an average CA of 86.13%, which is lower than the proposed method. On the other hand, “Spatially Sparse CSP” filters had been implemented by Arvaneh et al. [22]. SSCSP filters have emphasized that they have heavy weights within the area of the motor cortex. They reached a CA of 73.5%, which is 19.96% lower than our proposed system. In [65], the CSP approach was used for the training trials before assigning a score to each channel based on L1 norm scores. The authors in [46] extracted the RMS feature from the time domain only. An LDA classifier was built with 18 channels as well. They reached an average CA of 78.77%, which is lower than the proposed BCI system. Miao et al. [41] achieved a CA of 86.38% after extracting features depending on spatial-frequency-temporal patterns. The results suggest that R-CSP-A considerably outperforms the other methods concerning overall CA. Selim et al. reduced the number of CSP features and used the same 18 channels employed in [44] and introduced a hybrid feature selection model. J. Kervin et al. [48] achieved a CA of 92.8% using WPD and KNN classifier. In [66], the authors implemented a regularized-CSP with aggregation (R-CSP-A), in which a few R-CSPs are aggregated, providing an ensemble-based solution. They reached an average accuracy of 83.9%. The CSP approach was applied for feature extraction in [67]. A mutual information-based frequency band selection approach was proposed and got a mean CA of 91.68%. The results in Table S7 verify the competence performance of the proposed BCI system compared to other recent studies.

In Table S8, we compare the CA of the proposed BCI system and recent studies based on the autocalibration and recurrent adaptation dataset. The authors of [49] implemented an optimized system for rapid setup and fast co-adaptive training and reached CA 76.0%, which is about 10% lower than the result achieved by our proposed system. According to the data presented in Tables S7 and S8, the proposed BCI shows promising performance compared to recent studies. The performance of the proposed BCI to classify MI tasks with high accuracy is tested. The possibility of reducing the number of features and channels while improving classification accuracy is also examined. The proposed BCI results verified that the system has successfully reduced the number of channels and features while achieving a higher accuracy, which is greater than other recent related work, as shown in Tables S7 and S8 of the Supplementary Materials. Also, the system has avoided the limitations that existed in other related work. Thus, the proposed system may be considered an initial step for achieving a complete real-life--based framework to assist people with motor disability. Future work will apply the proposed system to individuals with limb motor disabilities to test their capability to improve their QoL.

## 6. Conclusions

This study proposes a novel efficient BCI system based on EEG data collected while performing MI tasks. The proposed system’s core objective was to develop a portable and low-cost BCI based on MI training neuro-feedback system to classify limb MI tasks in real time with high accuracy. This system is a preliminary stage for constructing a comprehensive real-life-based framework to aid people with motor deficiencies to make their daily activities. The proposed BCI system extracts features in the time-frequency domain for different sets of electrodes. It then fuses these features to form a hybrid feature set used to train SVM, LDA, and KNN classifiers. The results showed the proposed hybrid feature had increased the accuracy of the system. A hybrid feature extraction approach was also presented. This proposed FS lowered the computation cost and enhanced the BCI system’s accuracy for both the BCI competition III–IVa and the autocalibration and recurrent adaptation datasets. For the former dataset, the hybrid fused features using 18 channels after feature selection reached a mean CA of 93.56%, which is higher than most recent studies. For autocalibration and recurrent adaptation dataset, the hybrid fused features using 13 channels after feature selection yielded a mean CA of 86.41%, which is also higher than existing BCI systems. Our proposed systems’ competitive performance encourages the efficient usage of this system in future experiments to attain a comprehensive framework to assist people with motor disabilities in performing their daily functions. Future work will focus on testing the proposed BCI system’s capacity to improve individuals’ overall QoL with motor deficiencies. Also, upcoming research will investigate the system’s ability to successfully aiding people with wheelchairs or artificial limbs.

Further future work will investigate the use of the BCI system for rehabilitation procedures. Nevertheless, different experiments are still necessary to evaluate real-world rehabilitation treatments’ performance to test its performance on enhancing the rehabilitation treatments. Additional forthcoming work will focus on using the proposed BCI system in other applications such as driving, controlling a robot, communications, etc. New multiclass datasets containing more MI tasks should be used to test the proposed system’s performance.

## Figures and Tables

**Table 2:** Experiment 3—Hybrid features—Performance metrics for SVM classifier with channel set 2 (18 channels) for BCI competition III–IVa dataset.

Subjects	“aa”	“al”	“av”	“aw”	“ay”	Mean (SD)
**Sensitivity**	0.907	1.00	0.736	0.964	0.921	0.9056 (0.091)
**Specificity**	0.893	0.979	0.693	0.964	0.914	0.8886 (0.097)
**Precision**	0.894	0.979	0.705	0.964	0.915	0.8914(0.098)
**F1-score**	0.901	0.989	0.720	0.964	0.918	0.8984(0.095)
**AUC**	0.930	1.00	0.830	1.00	0.980	0.948(0.064)

**Table 3:** Experiment 3—Hybrid features—Performance metrics for SVM classifier with channel set 1 Auto (13 channels) for the autocalibration and recurrent adaptation dataset.

Subjects	“S01”		“S02”		“S03”		Mean (SD)
Runs	S01A	S01B	S02A	S02B	S03A	S03B	
Sensitivity	0.936	0.914	0.879	0.879	0.871	0.871	0.892 (0.025)
Specificity	0.929	0.900	0.864	0.864	0.864	0.871	0.882 (0.024)
Precision	0.929	0.901	0.866	0.866	0.865	0.871	0.883 (0.024)
F1-Score	0.918	0.900	0.864	0.864	0.852	0.871	0.878 (0.023)
ROC Area	0.99	0.99	0.99	0.99	0.99	0.99	0.99 (0)

**Table 4:** Experiment 4—The Hybrid Feature Selection using the channel set 2 BCI (18 channels) for the BCI competition III–IVa dataset.

Subjects	aa	al	av	aw	ay	CA	SD
Linear-SVM	92.2	99.4	79.9	98.9	97.0	93.46	8.1
LDA	83.93	97.15	71.93	93.22	87.4	86.73	9.72
K-NN	82.1	98.6	74.3	96.4	92.1	88.7	10.24

**Table 5:** Experiment 4—The Hybrid Feature Selection’ performance metrics of the SVM classifier constructed with the channel set 2 BCI (18 channels) for the BCI competition III–IVa dataset.

Subjects	“aa”	“al”	“av”	“aw”	“ay”	Mean (SD)
Sensitivity	0.914	1.00	0.786	0.993	0.950	0.9286 (0.0778)
Specificity	0.907	0.986	0.779	0.993	0.935	0.92 (0.0774)
Precision	0.901	0.986	0.780	0.993	0.937	0.9194 (0.0774)
F-Measure	0.931	0.993	0.783	0.993	0.937	0.9274(0.0769)
ROC Area	0.933	0.993	0.782	0.993	0.943	0.9288(0.7747)

**Table 6:** Experiment 4—The Hybrid Feature Selection CA using the Channel Set 1 Auto (13 channels) for the autocalibration and recurrent adaptation dataset.

Classifier	Runs	S01	S02	S03	S04	S05	S06	S07	S08	S09	S10	S11	S12	CA (SD)
SVM	1st	99.0	97.0	97.0	90.0	94.5	77.0	89.0	66.5	94.0	81.0	92.0	64.5	
	2nd	99.5	97.5	96.0	92.0	87.5	83.0	95.0	75.0	88.0	70.0	75.0	66.5	
	3rd	--	--	--	---	--	--	--	81.0	96.0	71.5	81.5	--	
Mean for each subject		99.25	97.25	96.5	91.0	91.0	80.0	92.0	74.17	92.7	74.7	82.83	65.5	86.41 (10.31)
LDA	1st	99.0	96.5	96.5	88.0	94.0	76.5	88.5	65.5	91.5	80.5	90.0	63.5	
	2nd	99.5	96.5	95.0	93.0	85.0	81.5	94.0	75.0	87.0	69.0	73.5	66.0	
	3rd	--	--	--	--	--	--	--	81.0	97.0	70.5	79.5	--	
Mean for each subject		99.25	96.5	95.75	90.5	89.5	79.0	91.25	73.83	91.83	73.33	81.0	64.75	85.54 (10.44)
KNN	1st	99.5	96.5	96.5	88.5	94.0	77.0	88.5	66.0	93.0	80.0	91.0	64.0	
	2nd	99.5	97.0	96.0	93.0	86.0	82.0	94.5	75.0	87.5	70.0	74.0	65.0	
	3rd	--	--	--	---	--	--	--	81.5	96.5	71.0	81.0	--	
Mean for each subject		99.5	97.25	96.25	90.75	90.0	79.5	91.5	74.17	92.33	73.67	82.0	64.5	85.95 (10.54)

**Table 7:** Experiment 4—Hybrid Feature Selection—Performance Metrics of SVM classifier constructed with Channel Set 1 Auto (13 channels) for autocalibration and recurrent adaptation dataset.

Subjects	“S01”		“S02”		“S03”		Mean (SD)
Runs	S01A	S01B	S02A	S02B	S03A	S03B	
Sensitivity	0.957	0.914	0.893	0.886	0.879	0.879	0.9058 (0.0279)
Specificity	0.950	0.907	0.900	0.871	0.879	0.879	0.9014 (0.0277)
Precision	0.950	0.907	0.899	0.873	0.879	0.879	0.9016 (0.0269)
F1-Score	0.950	0.907	0.896	0.869	0.879	0.879	0.9 (0.0271)
ROC Area	1.00	1.00	1.00	1.00	0.99	0.99	0.998 (0.0049)

**Table 8:** The number of features and execution time after the proposed Hybrid feature selection for the BCI competition III–IVa and the autocalibration and recurrent adaptation datasets.

Dataset	Features of the Full Set	Features after FS	Ex. Time before FS (s)	Ex. Time after FS (s)
BCI competition III–IVa dataset	1836	6	13.1964	1.2694
Autocalibration and recurrent adaptation	1326	9.679	12.6015	1.8571

**Figure A1:**
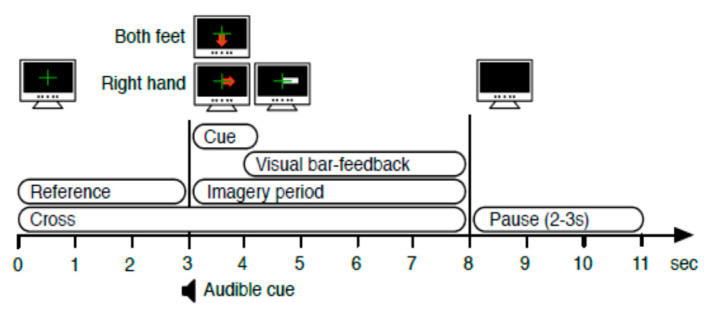
Experiment Paradigm [49].

## Supplementary Materials

The following are available online at https://www.mdpi.com/2076-3425/10/11/864/s1. Table S1: Experiment 1—Time domain features—Classification Accuracies Results for every subject of BCI competition III–IVa dataset Table S2: Experiment 1—Time domain features—Classification Accuracies (%) for every subject of the autocalibration and recurrent adaptation dataset. Table S3: Experiment 2–Time-Frequency domain—Classification Accuracies for every subject of the BCI competition III–IVa dataset. Table S4: Experiment 2—Time-Frequency domain–Classification Accuracies Results for every subject of the autocalibration and recurrent adaptation dataset. Table S5: Experiment 3—Hybrid features—Classification Accuracies for every subject of the BCI competition III–IVa dataset. Table S6: Experiment 3—Hybrid features—Classification Accuracies for every subject of the autocalibration and recurrent adaptation dataset. Table S7: A comparison between the CA of the proposed BCI system and recent related studies based on the BCI competition III–IVa dataset. Table S8: A comparison between the CA of the proposed BCI system and recent associated studies based on autocalibration and recurrent adaptation dataset.

## Appendix A

The BCI competition III–IVa dataset was collected from five healthy subjects (aa, al, av, aw, and ay). They were first seated in a comfortable chair. Then, EEG signals were acquired using 118 channels for the five subjects. Subjects were asked to perform MI tasks during EEG recording. MI tasks consisted of right-hand movement (RH) and foot movement (F). Visual signs indicated the type of MI task that each subject was to perform for 3.5 s. The number of such signs was 280. Short breaks of around 2 s were given to subjects between each successive visual sign. Electrodes were attached according to the international 10–20 system to describe the location of scalp electrodes. A bandpass filter was then applied to EEG signals from 0.05 Hz to 200 Hz. Signals were then digitized with a sampling rate of 1000 Hz then down-sampled to 100 Hz. 100 Hz signals were used in this study. The trials were unevenly split into training and evaluation trials for each subject, as shown in Table A1.

**Table A1:** Training and test trials for each person.

Person	Training Trials	Evaluation of Trials
**“aa”**	168	112
**“al”**	224	56
**“av”**	84	196
**“aw”**	56	224
**“ay”**	28	252

## Appendix B

The autocalibration and recurrent adaptation dataset were recorded for 12 healthy participants. These participants included seven males and five females (ages 24.8 ± 3 years). Participants sat in a comfortable chair and asked to relax. Then, EEG signals were acquired for all participants while performing a MI task using 13 electrodes, including C3 (FC3, C5, CP3, and C1), Cz (FCz, C1, CPz, and C2) and C4 (FC4, C2, CP4, and C6). These MI tasks consisted of a right-hand movement (RH) and a foot movement (F). Electrodes were attached according to the international 10–20 system to describe the location of scalp electrodes. Signals were digitized at a sampling frequency of 512 Hz. Afterwards, artifacts were removed using a bandpass filter from 0.5 Hz to 100 Hz. For this dataset, a notch filter was applied to eject the 50 Hz power-line noise. Visual signs indicated the type of MI task that each participant was to perform for 5 s. The number of such signs was 200. Short breaks of around 3s were given to the subjects between each successive visual sign presentation. The trial structure is shown in Figure A1. Two electrode sets were chosen; the 13 channels (full set) available as in^27^ and the 3 channels set C3, Cz, and C4.
