# Supplementary material for: A BCI System Based on Motor Imagery for Assisting People with Motor Deficiencies in the Limbs
Source: Brain Sci. 2020 Nov 17;10(11):864. doi: 10.3390/brainsci10110864 (PMC7697603; doi:10.3390/brainsci10110864)
Supplement: Supplementary file 1 [file brainsci-10-00864-s001.zip › Table S5.docx]

**Table S5.** Experiment 3– Hybrid features – Classification Accuracies for every subject of the BCI competition III-IVa dataset.

| **channels** | **Channel Set 1 BCI** | | | **Channel Set 2 BCI** | | | **Channel Set 3 BCI** | | |
| --- | --- | --- | --- | --- | --- | --- | --- | --- | --- |
| **subjects** | **SVM** | **LDA** | **KNN** | **SVM** | **LDA** | KNN | SVM | LDA | KNN |
| **aa** | 72.1 | 71.1 | 70.4 | **89.6** | 74.6 | 78.6 | 87.5 | 77.8 | 78.6 |
| **al** | 93.6 | 90.7 | 94.3 | **99.3** | 96.4 | 96.8 | 77.9 | 97.4 | 98.5 |
| **av** | 69.6 | 65.7 | 64.3 | **77.9** | 67.5 | 68.6 | 76.1 | 69.9 | 68.3 |
| **aw** | 90.4 | 87.9 | 90.4 | 97.5 | 89.3 | 93.2 | **97.9** | 92.1 | 93.5 |
| **ay** | 90.4 | 81.1 | 85.4 | 94.3 | 85.7 | 85.7 | **96.1** | 87.1 | 90.5 |
| **Average** | **83.22** | 80.3 | 81.97 | **91.72** | 84.5 | 85.52 | **91.1** | 84.86 | 85.8 |
| **SD** | 11.4 | 10.77 | 13.04 | 8.56 | 11.75 | 11.4 | 7.8 | 11.04 | 12.33 |
