# Supplementary material for: A BCI System Based on Motor Imagery for Assisting People with Motor Deficiencies in the Limbs
Source: Brain Sci. 2020 Nov 17;10(11):864. doi: 10.3390/brainsci10110864 (PMC7697603; doi:10.3390/brainsci10110864)
Supplement: Supplementary file 1 [file brainsci-10-00864-s001.zip › Table S6.docx]

**Table S6.** – Experiment 3– Hybrid features – Classification Accuracies for every subject of the Autocalibration and Recurrent Adaptation Dataset.

| **Classifier** | **Run** | **S01** | **S02** | **S03** | **S04** | **S05** | **S06** | **S07** | **S08** | **S09** | **S10** | **S11** | **S12** | **Mean CA(SD)**  **All Subjects** |
| --- | --- | --- | --- | --- | --- | --- | --- | --- | --- | --- | --- | --- | --- | --- |
| **SVM** | **1st** | 99.5 | 96.0 | 95.5 | 87.0 | 93.5 | 75.0 | 88.5 | 65.5 | 92.0 | 79.5 | 90.0 | 62.5 |  |
|  | **2nd** | 98.5 | 96.0 | 95.5 | 91.0 | 85.0 | 82.5 | 93.5 | 74.0 | 87.0 | 68.0 | 73.5 | 64.0 |  |
|  | **3rd** | -- | -- | -- | -- | -- | -- | -- | 81.5 | 95.5 | 69.0 | 79.0 | -- |  |
| **Mean for each subject** | | **99.0** | **96.0** | **95.5** | 89.0 | **89.25** | **78.75** | **91.0** | **73.67** | **91.5** | **72.17** | **80.83** | **63.25** | **85.0(10.54)** |
| **LDA** | **1st** | 98.2 | 95.0 | 94.1 | 85.3 | 93.0 | 74.2 | 87.0 | 63.3 | 91.2 | 77.6 | 82.9 | 62.0 |  |
|  | **2nd** | 97.7 | 95.1 | 94.1 | 95.2 | 83.5 | 82.0 | 92.6 | 72.1 | 86.7 | 67.5 | 73.0 | 63.4 |  |
|  | **3rd** | -- | -- | -- | -- | -- | -- | -- | 80.8 | 93.5 | 68.0 | 77.5 | -- |  |
| **Mean for each subject** | | 97.95 | 95.05 | 94.1 | 90.25 | 88.25 | 78.1 | 89.8 | 72.07 | 90.47 | 71.03 | 77.8 | 62.7 | 83.96(11.23 |
| **KNN** | **1st** | 99.0 | 95.3 | 95.1 | 86.0 | 93.1 | 74.4 | 88.1 | 65.0 | 91.6 | 78.4 | 85.6 | 62.1 |  |
|  | **2nd** | 98.1 | 95.4 | 95.0 | 95.5 | 84.1 | 82.3 | 93.1 | 73.0 | 86.8 | 67.9 | 73.1 | 63.5 |  |
|  | **3rd** | -- | -- | -- | -- | -- | -- | -- | 81.1 | 95.0 | 68.3 | 78.6 | -- |  |
| **Mean for each subject** | | 98.55 | 95.35 | 95.05 | **90.75** | 88.6 | 78.35 | 90.6 | 73.03 | 91.13 | 71.53 | 79.1 | 62.8 | 84.57(11.28) |
| **L-SVM** | **1st** | 98.0 | 95.0 | 96.0 | 85.5 | 92.0 | 71.5 | 87.5 | 58.0 | 91.0 | 79.0 | 90.0 | 62.0 |  |
|  | **2nd** | 98.5 | 95.5 | 96.5 | 89 | 83.0 | 83.0 | 89.5 | 68.5 | 85.5 | 67.0 | 71.0 | 63.0 |  |
|  | **3rd** | -- | -- | -- | -- | -- | -- | -- | 73.0 | 96.0 | 69.0 | 78.0 | -- |  |
| **Mean for each subject** | | 96.75 | 94.25 | 95.25 | 85.5 | 85.75 | 73.0 | 88.5 | 66.5 | 90.33 | 69.33 | 79.67 | 62.5 | 82.76(11.85) |
| **LDA** | **1st** | 96.5 | 92.0 | 95.0 | 82.9 | 86.0 | 70.5 | 86.0 | 57.3 | 90.2 | 78.0 | 88.5 | 61.2 |  |
|  | **2nd** | 95.0 | 95.0 | 92.8 | 85.3 | 82.1 | 74.3 | 87.8 | 68.0 | 85.3 | 65.2 | 70.4 | 62.5 |  |
|  | **3rd** | -- | -- | -- | -- | -- | -- | -- | 72.0 | 95.0 | 66.8 | 77.1 | -- |  |
| **Mean for each subject** | | 95.25 | 93.0 | 94.4 | 84.6 | 84.5 | 72.2 | 87.4 | 65.77 | 90.17 | 70.0 | 78.67 | 61.85 | 81.48(11.58) |
| **KNN** | **1st** | 96.0 | 93.5 | 95.5 | 81.0 | 88.2 | 70.0 | 87.2 | 59.0 | 90.5 | 78.3 | 88.9 | 61.5 |  |
|  | **2nd** | 96.5 | 95.3 | 93.5 | 85.8 | 81.3 | 81.5 | 88.5 | 68.2 | 85.5 | 66.3 | 70.6 | 62.7 |  |
|  | **3rd** | -- | -- | -- | -- | -- | -- | -- | 72.1 | 95.3 | 67.1 | 77.5 | -- |  |
| **Mean for each subject** | | 96.0 | 93.9 | 94.75 | 85.25 | 84.75 | 72.6 | 87.85 | 66.43 | 90.43 | 70.57 | 79.0 | 62.1 | 81.97(11.66) |
