# Supplementary material for: A BCI System Based on Motor Imagery for Assisting People with Motor Deficiencies in the Limbs
Source: Brain Sci. 2020 Nov 17;10(11):864. doi: 10.3390/brainsci10110864 (PMC7697603; doi:10.3390/brainsci10110864)
Supplement: Supplementary file 1 [file brainsci-10-00864-s001.zip › Table S7.docx]

**Table S7.** Comparison with recent BCI systems based on BCI competition III-IVa dataset.

| **Article** | **Method** | **Accuracy (%)** | | | | | |
| --- | --- | --- | --- | --- | --- | --- | --- |
|  |  | **aa** | **al** | **av** | **aw** | **ay** | **Mean (%)** |
| [30] | CSP/AR/LDA | 96.0 | 100.0 | 81.0 | 100.0 | 98.0 | 94.20 |
| [54] | R-CSP | 76.80 | 98.20 | 74.50 | 92.90 | 77.0 | 83.90 |
| [52] | CSP + SVM | 82.40 | 98.60 | 76.80 | 94.0 | 96.6 | 89.68 |
| [53] | SSCSP | 72.32 | 96.42 | 54.10 | 70.53 | 73.51 | 73.50 |
| [34] | RMS+ LDA | 74.11 | 96.43 | 60.71 | 71.88 | 84.20 | 78.80 |
| [29] | Spatial-Frequency-Temporal Patterns | 81.25 | 100.0 | 65.31 | 93.30 | 92.06 | 86.38 |
| [32] | CSP\AM-BA-SVM | 86.61 | 100.0 | 66.84 | 90.63 | 80.95 | 85.01 |
| [31] | SR-MDRM | 79.46 | 100.0 | 73.46 | 89.28 | 88.49 | 86.13 |
| [35] | MSPCA + WPD + HOS + k-NN | 96.00 | 92.30 | 88.90 | 95.40 | 91.40 | 92.80 |
| [57] | Channel selection +SVM | 59.20 | 91.0 | 58.50 | 83.50 | 87.80 | 76.0 |
| [55] | DFBCSP | 91.21 | 98.86 | 75.95 | 96.79 | 95.57 | 91.68 |
| **Proposed work** | | **92.2** | **99.4** | **79.9** | **98.9** | **97.0** | **93.46** |
