# Supplementary material for: A BCI System Based on Motor Imagery for Assisting People with Motor Deficiencies in the Limbs
Source: Brain Sci. 2020 Nov 17;10(11):864. doi: 10.3390/brainsci10110864 (PMC7697603; doi:10.3390/brainsci10110864)
Supplement: Supplementary file 1 [file brainsci-10-00864-s001.zip › Table S8.docx]

**Table S8.** Comparison with recent BCI systems based on Autocalibration and recurrent adaptation dataset.

| **Classifier** | **Runs** | **S01** | **S02** | **S03** | **S04** | **S05** | **S06** | **S07** | **S08** | **S09** | **S10** | **S11** |  | **S12** | **Mean CA (%) (SD)** |
| --- | --- | --- | --- | --- | --- | --- | --- | --- | --- | --- | --- | --- | --- | --- | --- |
| [J. Faller](https://www.researchgate.net/profile/Josef_Faller) et al. [56] | 1st | 98.1 | 95.9 | 83.1 | 80.8 | 78.0 | 72.7 | 72.7 | 62.4 | 63.6 | 67.6 | 69.0 |  | 60.9 |  |
|  | 2nd | 98.8 | 98.8 | 85.3 | 83.5 | 80.4 | 77.5 | 74.7 | 66.5 | 72.0 | 68.6 | 59.0 |  | 59.5 |  |
|  | 3rd | -- | -- | -- | -- | -- | -- | -- | 86.5 | 80.0 | 65.3 | 70.6 |  | -- |  |
| Mean for each subject | | 98.4 | 97.4 | 84.2 | 82.2 | 79.2 | 75.1 | 73.7 | 71.8 | 71.9 | 67.1 | 66.2 |  | 60.4 | 76.0 (11.31) |
| This work | 1st | 99.0 | 97.0 | 97.0 | 90.0 | 94.5 | 77.0 | 89.0 | 66.5 | 94.0 | 81.0 | 92.0 |  | 64.5 |  |
|  | 2nd | 99.5 | 97.5 | 96.0 | 92.0 | 87.5 | 83.0 | 95.0 | 75.0 | 88.0 | 70.0 | 75.0 |  | 66.5 |  |
|  | 3rd | -- | -- | -- | --- | -- | -- | -- | 81.0 | 96.0 | 71.5 | 81.5 |  | -- |  |
| Mean for each subject | | **99.25** | **97.25** | **96.5** | **91.0** | **91.0** | **80.0** | **92.0** | **74.17** | **92.7** | **74.7** | **82.83** |  | **65.5** | **86.41** (10.31) |
