# Supplementary material for: A BCI System Based on Motor Imagery for Assisting People with Motor Deficiencies in the Limbs
Source: Brain Sci. 2020 Nov 17;10(11):864. doi: 10.3390/brainsci10110864 (PMC7697603; doi:10.3390/brainsci10110864)
Supplement: Supplementary file 1 [file brainsci-10-00864-s001.zip › List of tables.docx]

**Table S1.** Experiment 1 –Time domain features – Classification Accuracies Results for every subject of BCI competition III-IVa dataset.

Table S2: Experiment 1 –Time domain features – Classification Accuracies (%) for every subject of the Autocalibration and Recurrent Adaptation dataset.

Table S3: Experiment 2–Time-Frequency domain – Classification Accuracies for every subject of the BCI competition III-IVa dataset.

Table S4: Experiment2–Time-Frequency domain–Classification Accuracies Results for every subject of the Autocalibration and Recurrent Adaptation dataset.

Table S5: Experiment 3– Hybrid features – Classification Accuracies for every subject of the BCI competition III-IVa dataset.

Table S6: Experiment 3– Hybrid features – Classification Accuracies for every subject of the Autocalibration and Recurrent Adaptation Dataset.

Table S7: A comparison between the CA of the proposed BCI system and recent related studies based on the BCI competition III-IVa dataset.

Table S8: A comparison between the CA of the proposed BCI system and recent related studies based on Autocalibration and recurrent adaptation dataset
