# Supplementary material for: A BCI System Based on Motor Imagery for Assisting People with Motor Deficiencies in the Limbs
Source: Brain Sci. 2020 Nov 17;10(11):864. doi: 10.3390/brainsci10110864 (PMC7697603; doi:10.3390/brainsci10110864)
Supplement: Supplementary file 1 [file brainsci-10-00864-s001.zip › Table S1.docx]

| **Classifier** | **Subjects** | **Channel Set 1** | **Channel Set 2** | **Channel Set 3** |
| --- | --- | --- | --- | --- |
| **SVM** | **aa** | 70.0 | 76.4 | **78.9** |
|  | **al** | 86.4 | 93.6 | **96.1** |
|  | **av** | 68.2 | 71.1 | **72.9** |
|  | **aw** | 86.4 | 93.2 | **93.6** |
|  | **ay** | 87.9 | 86.8 | **90.0** |
|  | **Mean** | 79.78 | 84.22 | **86.3** |
|  | **SD** | 9.78 | 10.1 | 9.97 |
| **LDA** | **aa** | 68.3 | 74.4 | **78.0** |
|  | **al** | 85.1 | 91.6 | **93.2** |
|  | **av** | 66.9 | 68.9 | **70.5** |
|  | **aw** | 82.4 | 89.1 | **90.5** |
|  | **ay** | 85.0 | 84.7 | **89.2** |
|  | **Mean** | 77.54 | 81.4 | **84.28** |
|  | **SD** | 9.15 | 9.73 | 7.43 |
|  | **aa** | 69.3 | 77.7 | **78.3** |
|  | **al** | 85.5 | 92.7 | **95.2** |
| **KNN** | **av** | 67.3 | 70.6 | **71.9** |
|  | **aw** | 84.4 | 92.8 | **92.9** |
|  | **ay** | 86.7 | 85.6 | **89.0** |
|  | **Mean** | 78.64 | 83.92 | **84.46** |
|  | **SD** | 9.5 | 9.46 | 10.03 |
