# Supplementary material for: A BCI System Based on Motor Imagery for Assisting People with Motor Deficiencies in the Limbs
Source: Brain Sci. 2020 Nov 17;10(11):864. doi: 10.3390/brainsci10110864 (PMC7697603; doi:10.3390/brainsci10110864)
Supplement: Supplementary file 1 [file brainsci-10-00864-s001.zip › Table S2.docx]

**Table S2.** Experiment 1 –Time domain features – Classification Accuracies (%) for every subject of the Autocalibration and Recurrent Adaptation dataset.

| **Classifier** | **Run** | **S01** | **S02** | **S03** | **S04** | **S05** | **S06** | **S07** | **S08** | **S09** | **S10** | **S11** | **S12** | **Mean CA(SD)**  **All Subjects** |
| --- | --- | --- | --- | --- | --- | --- | --- | --- | --- | --- | --- | --- | --- | --- |
| **Channel Set 1Auto** | | | | | | | | | | | | | | |
| **SVM** | **1st** | 95.3 | 94.0 | 91.6 | 83.1 | 90.2 | 71.1 | 85.1 | 63.1 | 90.0 | 78.0 | 87.0 | 60.4 |  |
|  | **2nd** | 96.7 | 93.5 | 92.5 | 89.6 | 82.5 | 79.6 | 90.2 | 71.2 | 84.8 | 65.5 | 71.1 | 61.6 |  |
|  | **3rd** | -- | -- | -- | -- | -- | -- | -- | 78.0 | 93.5 | 65.5 | 77.5 | -- |  |
| **Mean CA for each subject** | | **96.0** | **93.75** | **92.05** | 86.35 | **86.35** | **75.35** | **87.65** | **70.77** | **89.43** | **69.67** | **78.35** | **61.0** | **82.23 (10.73)** |
| **LDA** | **1st** | 93.1 | 91.7 | 89.3 | 81.1 | 89.5 | 70.6 | 81.1 | 59.9 | 88.1 | 71.5 | 78.5 | 56.6 |  |
|  | **2nd** | 92.5 | 92.1 | 89.3 | 90.1 | 79.5 | 77.1 | 89.6 | 69.3 | 81.3 | 60.9 | 70.3 | 58.2 |  |
|  | **3rd** | -- | -- | -- | -- | -- | -- | -- | 77.1 | 89.3 | 62.3 | 71.8 | -- |  |
| **Mean CA for each subject** | | 92.8 | 91.9 | 89.3 | 85.6 | 84.5 | 73.85 | 85.35 | 68.77 | 86.23 | 64.9 | 73.53 | 57.4 | 79.51  (12.29) |
| **KNN** | **1st** | 94.6 | 92.5 | 90.1 | 82.3 | 89.8 | 70.8 | 83.5 | 61.4 | 91.2 | 75.9 | 85.9 | 58.6 |  |
|  | **2nd** | 93.7 | 92.5 | 90.6 | 92.3 | 80.7 | 78.5 | 89.8 | 69.7 | 84.7 | 63.1 | 72.1 | 59.9 |  |
|  | **3rd** | -- | -- | -- | -- | -- | -- | -- | 77.8 | 92.7 | 62.9 | 75.1 | -- |  |
| **Mean CA for each subject** | | 94.15 | 92.5 | 90.35 | **87.3** | 85.25 | 74.65 | 86.65 | 69.63 | 89.53 | 67.3 | 77.7 | 59.25 | 80.94 (11.31) |
| **Channel Set 2 Auto** | | | | | | | | | | | | | | |
| **SVM** | **1st** | 94.9 | 93.0 | 90.1 | 81.5 | 89.1 | 69.3 | 83.2 | 60.5 | 89.3 | 77.5 | 86.3 | 58.9 | 58.9 |
|  | **2nd** | 96.1 | 93.0 | 89.1 | 87.3 | 80.1 | 77.2 | 88.2 | 70.0 | 82.8 | 65.0 | 70.2 | 60.0 | 60.0 |
|  | **3rd** | -- | -- | -- | -- | -- | -- | -- | 77.2 | 91.5 | 64.1 | 76.5 | -- | -- |
| **Mean CA for each subject** | | **95.5** | **93.0** | **89.6** | 84.4 | **84.6** | **75.25** | **85.7** | **69.23** | **87.87** | **68.87** | **77.67** | **59.45** | **80.93 (11.89)** |
| **LDA** | **1st** | 92.5 | 91.0 | 87.5 | 78.3 | 87.3 | 68.3 | 78.3 | 55.9 | 86.9 | 70.2 | 76.1 | 53.9 | 53.9 |
|  | **2nd** | 91.0 | 91.1 | 86.9 | 88.6 | 76.5 | 74.9 | 85.6 | 67.6 | 79.4 | 59.2 | 69.1 | 56.8 | 56.8 |
|  | **3rd** | -- | -- | -- | -- | -- | -- | -- | 76.8 | 87.6 | 59.9 | 70.0 | -- | -- |
| **Mean CA for each subject** | | 91.75 | 91.05 | 87.2 | 83.45 | 81.9 | 71.6 | 81.95 | 66.77 | 84.63 | 63.1 | 71.73 | 55.35 | 77.54 (13.2) |
| **KNN** | **1st** | 94.0 | 91.8 | 87.7 | 81.5 | 88.6 | 68.4 | 82.5 | 60.2 | 90.5 | 75.0 | 84.3 | 57.2 | 57.2 |
|  | **2nd** | 93.5 | 92.1 | 87.9 | 90.7 | 78.4 | 77.9 | 88.5 | 67.6 | 81.1 | 62.2 | 71.1 | 59.0 | 59.0 |
|  | **3rd** | -- | -- | -- | -- | -- | -- | -- | 76.2 | 90.8 | 62.0 | 73.5 | -- | -- |
| **Mean CA for each subject** | | 93.75 | 91.95 | 87.8 | **86.1** | 83.5 | 73.15 | 85.5 | 68.0 | 87.47 | 66.4 | 76.3 | 58.1 | 79.84 (10.93) |
