# Supplementary material for: A BCI System Based on Motor Imagery for Assisting People with Motor Deficiencies in the Limbs
Source: Brain Sci. 2020 Nov 17;10(11):864. doi: 10.3390/brainsci10110864 (PMC7697603; doi:10.3390/brainsci10110864)
Supplement: Supplementary file 1 [file brainsci-10-00864-s001.zip › Table S3.docx]

**Table S3 .** Experiment 2–Time-Frequency domain – Classification Accuracies for every subject of the BCI competition III-IVa dataset

| **Classifier** | **Channels**  **Subjects** | **Channel Set 1 BCI** | **Channel Set 2 BCI** | **Channel Set 3 BCI** |
| --- | --- | --- | --- | --- |
| **SVM** | **aa** | 70.4 | **84.6** | 86.4 |
|  | **al** | 91.4 | **98.9** | 98.9 |
|  | **av** | 68.2 | **77.9** | 69.3 |
|  | **aw** | 90.7 | **97.1** | 96.4 |
|  | **ay** | 88.6 | **93.2** | 95.4 |
|  | **Mean** | 81.86 | **90.34** | 89.28 |
|  | **SD** | 23.02 | **17.72** | 24.1 |
| **LDA** | **aa** | 69.2 | **76.9** | 80.1 |
|  | **al** | 86.5 | **94.3** | 90.7 |
|  | **av** | 67.1 | **70.9** | 67.2 |
|  | **aw** | 83.3 | **91.2** | 90.8 |
|  | **ay** | 86.7 | **88.0** | 90.1 |
|  | **Mean** | 78.56 | **84.26** | 83.78 |
|  | **SD** | 19.23 | **16.7** | 20.9 |
|  | **aa** | 70.1 | **80.1** | 82.6 |
|  | **al** | 87.8 | **95.6** | 95.7 |
| **KNN** | **av** | 68.0 | **72.9** | 68.0 |
|  | **aw** | 85.2 | **93.7** | 93.0 |
|  | **ay** | 87.7 | **90.0** | 90.3 |
|  | **Mean** | 79.76 | **86.84** | 85.92 |
|  | **SD** | 19.67 | **15.3** | 21.1 |
