# Supplementary material for: A BCI System Based on Motor Imagery for Assisting People with Motor Deficiencies in the Limbs
Source: Brain Sci. 2020 Nov 17;10(11):864. doi: 10.3390/brainsci10110864 (PMC7697603; doi:10.3390/brainsci10110864)
Supplement: Supplementary file 1 [file brainsci-10-00864-s001.zip › Table S4.docx]

**Table S4.** Experiment2–Time-Frequency domain–Classification Accuracies Results for every subject of the Autocalibration and Recurrent Adaptation dataset.

| **Classifier** | **Run** | **S01** | **S02** | **S03** | **S04** | **S05** | **S06** | **S07** | **S08** | **S09** | **S10** | **S11** | **S12** | **Mean CA(SDAll Subjects** |
| --- | --- | --- | --- | --- | --- | --- | --- | --- | --- | --- | --- | --- | --- | --- |
| **SVM** | **1st** | 97.2 | 95.0 | 93.3 | 85.2 | 91.9 | 74.2 | 88.0 | 64.2 | 90.8 | 78.3 | 88.2 | 61.7 |  |
|  | **2nd** | 97.0 | 95.0 | 91.8 | 90.7 | 83.8 | 80.8 | 91.2 | 72.3 | 85.5 | 66.9 | 72.0 | 62.7 |  |
|  | **3rd** | -- | -- | -- | -- | -- | -- | -- | 80 | 94.6 | 68.1 | 78.0 | -- |  |
| **Mean CA for each subject** | | **97.1** | **95.0** | **92.55** | **87.95** | **87.85** | **77.5** | **89.6** | **72.17** | **90.3** | **71.1** | **79.4** | **62.2** | **83.56 (11.68)** |
| **LDA** | **1st** | 95.1 | 93.6 | 92.1 | 83.2 | 92.1 | 72.1 | 83.3 | 61.2 | 89.9 | 71.9 | 80.8 | 58.8 |  |
|  | **2nd** | 94.5 | 93.2 | 91.2 | 90.0 | 80.5 | 80.5 | 91.1 | 70.0 | 83.1 | 64.7 | 71.2 | 61.1 |  |
|  | **3rd** | -- | -- | -- | -- | -- | -- | -- | 78.5 | 91.6 | 65.4 | 74.3 | -- |  |
| **Mean CA for each subject** | | 94.8 | 93.4 | 91.65 | 86.6 | 85.95 | 76.3 | 87.2 | 69.9 | 88.2 | 65.4 | 75.43 | 59.9 | 81.1  (11.07) |
| **KNN** | **1st** | 96.7 | 94.2 | 92.9 | 84.4 | 92.1 | 73.4 | 85.9 | 63.4 | 90.5 | 77.0 | 85.7 | 61.7 |  |
|  | **2nd** | 97.0 | 94.6 | 91.8 | 90.3 | 82.5 | 81.2 | 92.3 | 71.2 | 85.5 | 65.2 | 72.6 | 62.1 |  |
|  | **3rd** | -- | -- | -- | -- | -- | -- | -- | 79.0 | 94.1 | 67.5 | 76.6 | -- |  |
| **Mean CA for each subject** | | 96.85 | 94.4 | 92.2 | 87.35 | 87.3 | 77.3 | 89.1 | 71.2 | 90.37 | 69.9 | 78.3 | 61.9 | 83.33 (10.8) |
| **SVM** | **1st** | 96.3 | 94.1 | 92.0 | 83.2 | 90.7 | 70.4 | 85.5 | 59.8 | 90.7 | 78.0 | 87.7 | 61.4 |  |
|  | **2nd** | 96.5 | 94.2 | 91.0 | 88.5 | 82.1 | 79.3 | 89.0 | 70.1 | 84.6 | 66.6 | 70.9 | 62.2 |  |
|  | **3rd** | -- | -- | -- | -- | -- | -- | -- | 72.9 | 94.2 | 67.3 | 77.3 | -- | -- |
| **Mean CA for each subject** | | **96.4** | **94.15** | **91.5** | **85.85** | **86.4** | **74.85** | **87.25** | **67.6** | **89.83** | **70.63** | **78.63** | **61.8** | **82.06 (10.7)** |
| **LDA** | **1st** | 94.4 | 91.5 | 91.9 | 82.1 | 87.5 | 69.6 | 82.8 | 56.3 | 87.0 | 71.8 | 79.8 | 57.2 |  |
|  | **2nd** | 93.2 | 92.2 | 90.3 | 88.2 | 80.0 | 75.0 | 86.7 | 67.8 | 82.8 | 62.6 | 70.2 | 60.4 |  |
|  | **3rd** | -- | -- | -- | -- | -- | -- | -- | 71.9 | 91.3 | 63.9 | 73.5 | -- |  |
| **Mean CA for each subject** | | 93.8 | 91.85 | 91.1 | 85.1 | 83.75 | 72.3 | 84.75 | 67.07 | 87.03 | 66.1 | 74.5 | 58.8 | 79.76 (11.11) |
| **KNN** | **1st** | 95.1 | 92.5 | 88.0 | 82.2 | 89.0 | 70.1 | 84.9 | 59.0 | 90.5 | 76.6 | 85.2 | 61.1 |  |
|  | **2nd** | 95.1 | 93.9 | 90.7 | 88.4 | 82.0 | 79.0 | 88.3 | 68.0 | 83.5 | 65.5 | 71.0 | 61.7 |  |
|  | **3rd** | -- | -- | -- | -- | -- | -- | -- | 72.2 | 93.5 | 66.5 | 75.2 | -- |  |
| **Mean CA for each subject** | | 95.1 | 93.2 | 89.5 | 85.3 | 85.5 | 74.55 | 86.6 | 66.4 | 89.17 | 69.53 | 77.13 | 61.4 | 80.88 (10.87) |
